# The Reduced Expression of EOLA1 May Be Related to Refractory Diabetic Foot Ulcer

**DOI:** 10.1155/2019/6705424

**Published:** 2019-03-17

**Authors:** Mingxia Wu, Weiling Leng, Hang Pan, Xiaotian Lei, Liu Chen, Xinshou Ouyang, Ziwen Liang

**Affiliations:** ^1^Health Management Center, First Affiliated Hospital of Army Medical University (Third Military Medical University), Chongqing 400038, China; ^2^Department of Endocrinology, First Affiliated Hospital of Army Medical University (Third Military Medical University), Chongqing 400038, China; ^3^Department of Internal Medicine, Section of Digestive Diseases, Yale University of Medicine, New Haven, CT 06520, USA

## Abstract

**Background:**

Chronic diabetic foot ulcer (DFU) is one of the most intractable complications of diabetes mellitus (DM). Its pathogenesis is complex, and uncontrolled chronic inflammation is an important factor. Endothelial overexpressed lipopolysaccharide-associated factor 1 (EOLA1) discovered in our laboratory is an intracellular protein with the function of inflammatory regulation. This study was aimed at observing the expression of EOLA1 in DFU skin tissues and its relationship with inflammation and at exploring the possible role of EOLA1 in DFU and its mechanism.

**Methods:**

The patients with DFU were divided into 2 groups based on the formation time of ulcer: the acute wound (AW) group with the course of disease ≤ 4 weeks and the chronic wound (CW) group with the course of disease > 4 weeks. The relevant clinical data of patients were collected, and the skin tissues around the ulcer were used for immunofluorescence detection and immunohistochemical staining to observe inflammation. The expression levels of EOLA1, metallothionein 2A (MT2A), nuclear factor-*κ*B (NF-*κ*B), and interleukin-6 (IL-6) were detected by western blot.

**Results:**

A total of 79 patients were enrolled in the study. The results of immunofluorescence and immunohistochemistry showed that EOLA1 was expressed in the epithelial tissues of DFU. However, the expression of EOLA1 in the CW group was significantly lower than that in the AW group (*P* < 0.05), and the expression of NF-*κ*B and IL-6 was obviously increased (*P* < 0.05).

**Conclusion:**

The refractory wounds in patients with DFU may be closely related to the uncontrolled activation of inflammatory pathways in cells caused by the reduced expression of negative regulators of inflammation (e.g., EOLA1), and such decreased expression may be also strongly linked to the persistent state of inflammation.

## 1. Introduction

With the high rates of disability and death, chronic diabetic foot ulcer (DFU) is one of the most refractory complications of diabetes mellitus (DM), and it seriously affects the patients' quality of life and life expectancy [1]. The main cause for DFU is a protracted course of inflammation in wounds, which is manifested as reduced apoptosis of inflammatory cells, abnormal phenotypic transformation of macrophage M1/M2, and failure to timely terminate the inflammatory signaling pathway of intracellular activated nuclear factor-*κ*B (NF-*κ*B). However, the specific pathogenesis of DFU has not been completely clarified and the treatment effect is poor [2]. Although the clinical measures are widely taken, such as debridement, decompression, antibacterial therapy, neurotrophic improvement, and revascularization, the risk of amputation remains high in patients with DFU [3]. In the recent years, new techniques including autologous platelet-rich gel [4] and marrow stem cells [5, 6] have achieved good effects in treating chronic wounds of DFU. In spite of the nonelucidated mechanism, the inhibition of local inflammatory immune response is an important part in treating DFU.

Endothelial overexpressed lipopolysaccharide-associated factor 1 (EOLA1) is a gene with unknown functions which is cloned from endotoxin-stimulated human vascular endothelial cells by suppression subtractive hybridization in our laboratory [7]. The EOLA1 gene is located at human chromosome Xq28, and the secondary structure of EOLA1 contains the phosphorylation sites of protein kinase C and tyrosine kinase, as well as helix-turn-helix (HTH) motifs, which are located in cells and participate in the intracellular signal transduction process as transcription factors. EOLA1 is weakly expressed in leukocytes and endothelial cells under a resting state, and such expression can be significantly increased after stimulation with lipopolysaccharide (LPS). EOLA1 has the functions of promoting cell growth, inhibiting apoptosis, and downregulating the secretion of immune-inflammatory factors such as intracellular interleukin-6 (IL-6) and intercellular adhesion molecule-1 (ICAM-1) [8, 9]. In addition, EOLA1 is associated with cell growth, and the growth of ECV304 cells is significantly slowed down after the inhibition of EOLA1 expression [10].

Metallothionein (MT) is a class of cysteine-rich intracellular protein with a low molecular weight (6~7 kDa). MT participates in the detoxification of heavy metals, scavenging of free radicals, inhibition of lipid peroxidation, antagonism of ionizing radiation, and light protection [11]. It has been found that there are two subtypes of MT in skin tissues, that is, MT-I and MT-II [12]. The previous studies have shown that the expression level of MT in skin tissues is decreased with age [13], and the mouse skin lacking MT is more vulnerable to UV damage [14], which suggests that MT may play an important role in the growth and repair of skin tissues. Our preliminary study demonstrated that EOLA1 participated in the regulation of intracellular inflammation-related signaling pathways by the interaction with metallothionein 2A (MT2A) in cells [7]. The persistent inflammatory response is an important factor in the refractory wounds of patients with DFU, but the role and importance of EOLA1 in this process are not clear. In this study, in order to explore the potential anti-inflammatory and prohealing effects of EOLA1 in DFU and its possible mechanism, the tissue samples of the acute wound (AW) and chronic wound (CW) in DFU were collected to detect the expression of EOLA1 in the skin tissues of DFU patients at different stages and analyze the relationship between the expression changes of EOLA1 and MT2A and the wound inflammatory response.

## 2. Methods

### 2.1. Case Collection

The patients with DFU who were hospitalized in the Diabetic Foot Center of the First Affiliated Hospital of Army Medical University (Third Military Medical University) were selected for the study. Type 2 DM was diagnosed according to the WHO diagnostic criteria in 1999. The inclusion criteria were as follows: age>18 years, Wagner 2-5, no obvious systemic clinical infection, ankle-to-brachial ratio index (ABI) > 0.9, wound size = 2-25 cm^2^, and glycosylated hemoglobin (HbA1c) ≤ 14%. The exclusion criteria were described as follows: Wagner ≤ 1, ABI ≤ 0.9, HbA1c > 14%, severe systemic infection, suspected wound with tumor formation, or tuberculosis. The preexperiment was performed on the first 10 patients separately enrolled into two groups, and the expression level of EOLA1, as preexperimental data, was calculated by a statistic formula which indicated that the sample size in each group needs no less than 10. Ultimately, a total of 79 patients were enrolled in the study, including 38 patients with early DFU (ulcer formation time ≤ 4 weeks) and 41 patients with advanced DFU (ulcer formation time > 4 weeks). Before the patients were administrated with antibiotics after admission, the ulcer was rinsed with normal saline, followed by debridement; then, the wound and surrounding tissues 0.5 cm away from the wound margin were resected with sterile scalpels for examination. At the same time, the basic information of patients and the DM-related complications were collected. All patients signed the informed consent forms. The study was approved by the Ethics Committee of the First Affiliated Hospital of Army Medical University.

### 2.2. Collection of Clinical Data

After admission, the diabetic feet of patients were evaluated according to the Wagner classification standard [15]. The blood samples were collected via the median cubital vein and sent to the laboratory of Southwest Hospital for the routine detection of biochemical indicators, such as HbA1c, high-sensitivity C-reactive protein (HsCRP), procalcitonin (PCT), IL-6, white blood cells (WBC), hemoglobin (HGB), neutrophil percentage (NE%), and albumin (Alb). A special instrument was used to measure the ankle-brachial index (ABI) of the suffered limb. After ulcer rinsing with normal saline and debridement, the infected tissues were collected with a sterilized tube and then rapidly sent to the microbiological laboratory of the laboratory department for microbial culture and drug sensitivity test. The patients were enrolled according to the inclusion criteria and exclusion criteria.

### 2.3. Pathological Examination of Wound Skin Tissues

The wound skin tissues were fixed in 4% paraformaldehyde overnight at 4°C, dehydrated, vitrified, embedded, and cut into paraffin sections with a thickness of 4 *μ*m. Then, the sections were stained with HE and observed under a light microscope. After baking and dewaxing treatment, the paraffin sections were kept in 3% H_2_O_2_ for 20 min and then in 10 mmol/L sodium citrate buffer (pH 6.0) for 4 min within a microwave oven at 92-98°C. Subsequently, they were blocked with goat serum, incubated with primary antibodies (rabbit anti-human EOLA1 polyclonal antibody, 1 : 50, NOVUS; rabbit anti-human IL-6 polyclonal antibody, 1 : 200, Abcam; mouse anti-human MT2A monoclonal antibody, 1 : 20, Abcam; and rabbit anti-human NF-*κ*B P65 polyclonal antibody, 1 : 500, Abcam) overnight at 4°C, and then incubated with secondary antibodies at room temperature for 30 min. After coloration with DAB, counterstaining with hematoxylin, and dehydration, these sections were mounted with gum. The images were analyzed using Image-Pro Plus 6.0 software after observation and photography under a light microscope, and the average optical density (OD) was statistically analyzed.

### 2.4. Immunofluorescence Detection of EOLA1 Expression in Skin Tissues

After OCT embedding, the wound skin tissues were put into the freezing microtome, then cut into sections with a thickness of 4 *μ*m and fixed on glass slides. Next, they were fixed in 4% paraformaldehyde, blocked with goat serum and incubated with a primary antibody (rabbit anti-human EOLA1 polyclonal antibody, 1 : 1000, Abcam) overnight at 4°C, and then incubated with a secondary antibody for 30 min. After staining with DAPI, they were mounted with antifade mounting medium and immediately observed under a laser scanning confocal microscope. The images were analyzed using ImageJ software after observation and photography, and the average optical density (OD) was statistically analyzed.

### 2.5. Western Blot

The skin tissues cytopreserved in liquid nitrogen were ground in a mortar, added with protein lysis buffer and protease inhibitor, then ground again with the ultrasonic homogenizer, and centrifuged. The supernatant was collected, added with bromophenol blue to boil, and cooled. Thereafter, the proteins in tissues were quantitatively analyzed. They were incubated with a primary antibody (rabbit anti-human EOLA1 polyclonal antibody, 1 : 1000, Biorbyt) overnight at 4°C after electrophoresis and membrane transfer and then incubated with a secondary antibody for 2 h. The expression of EOLA1, NF-*κ*B, IL-6, and MT2A was detected with Image Lab software.

### 2.6. Statistical Analysis

All data were analyzed using the SPSS 19.0 software. The measurement data were expressed as the mean ± standard deviation ($\bar{x}\pm s$) and analyzed using the independent *t*-test. The counting data were analyzed with the chi-square test with a significance level of *α* = 0.05.

## 3. Results

### 3.1. Basic Information of Patients

A total of 79 patients were enrolled in the study, including 20 females and 59 males, and there was no difference in the sex ratio between the two groups (*χ*^2^ = 0.70, *P* = 0.40). The age of patients ranged from 37 to 88 years; the average age in the acute wound (AW) group was 62.61 ± 12.31 years, and that in the chronic wound (CW) group was 65.95 ± 11.25 years. The hospitalization time of patients in the CW group was significantly prolonged, and the proportion of patients requiring surgical treatment was increased (46.34%), but the surgical methods (debridement, use of polymethylmethacrylate, digital or ray amputation, and transtibial amputation) between the two groups were undifferentiated (*χ*^2^ = 0.38, *P* = 0.54). There was no obvious difference between the two groups in the positive rate of bacterial culture, the ratio of gram-positive to gram-negative bacteria, and the incidence rate of diabetic complications such as vascular diseases, neuropathy, and retinopathy. In addition, no statistically significant difference was observed in the plasma inflammatory indicators, HsCRP (*t* = 0.25, *P* = 0.80), PCT (*t* = 1.48, *P* = 0.16), IL-6 (*t* = 0.04, *P* = 0.97), WBC (*t* = −0.54, *P* = 0.09), and NE% (*t* = 0.41, *P* = 0.68). The proportion of patients with better blood glucose control (HbA1c < 8%) was higher in the CW group (41.46%), but there was no statistical difference in the distribution of HbA1c (*χ*^2^ = 1.35, *P* = 0.25), as well as HGB (*t* = 1.14, *P* = 0.16), Alb (*t* = 0.41, *P* = 0.68), and ABI (*χ*^2^ = 2.56, *P* = 0.28) (Table 1).

### 3.2. Histological Analysis of DFU Skin Tissues

**Figure 1:**
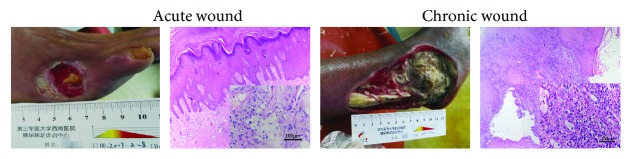
The condition of the diabetic foot wound and the inflammatory cellular infiltration (HE staining, ×200).

In DFU, the acute wound was bright red and had obvious granulation. The results of HE staining indicated that the infiltration of inflammatory cells was significant in the surrounding skin tissues. The chronic wound was dark and only had granulation in a few of peripheral skin tissues. Moreover, HE staining showed a large number of inflammatory cells infiltrating the surrounding skin tissues (Figure 1).

### 3.3. Expression of EOLA1

**Figure 2:**
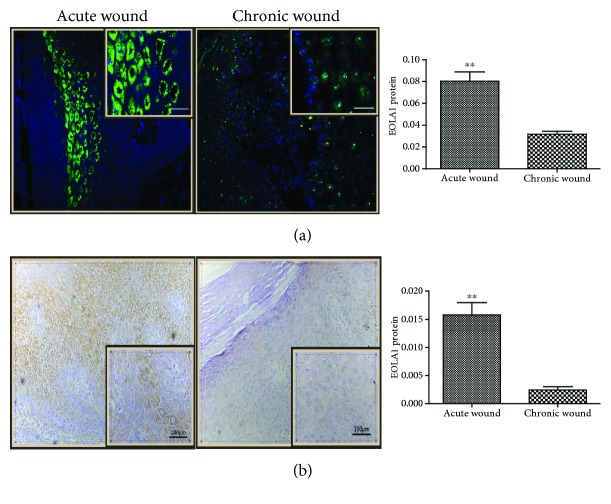
EOLA1 expression in the epidermis of diabetic foot skin tissues. The expression of the chronic wound was obviously decreased. (a) EOLA1 protein (green) identified by immunofluorescence. Cell nuclei were detected by DAPI (blue). Scale bar: 100 *μ*m. (b) Immunohistochemical analysis of EOLA1 in the diabetic foot skin tissues. The yellowish-brown color denotes the positive areas. The quantitative analysis of EOLA1 expression was performed through measuring the average optical density. ^∗∗^*P* < 0.01.

The results of immunofluorescence detection revealed that EOLA1 was significantly expressed in the cytoplasm of epithelial squamous cells within the AW group, and the expression was obviously decreased in the CW group (*t* = 5.476, *P* < 0.001). Furthermore, the results obtained from the immunohistochemical assay showed high expression of EOLA1 in the cytoplasm of squamous epithelial cells near the basal layer within the AW group but weak expression within the CW group. The average ODs of the two groups demonstrated a statistically significant difference (*t* = 4.291, *P* = 0.001) (Figure 2).

### 3.4. Immunohistochemical Analysis on the Expression of NF-*κ*B, IL-6, and MT2A

**Figure 3:**
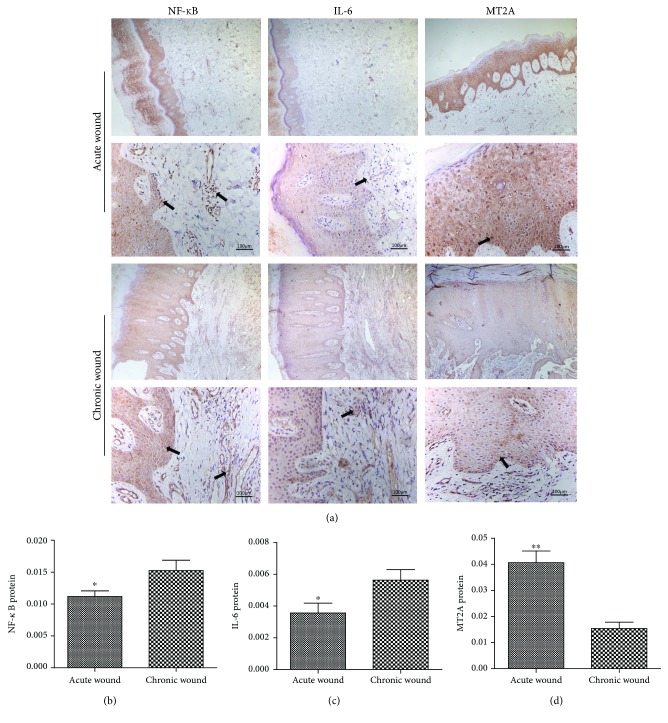
Expression of NF-*κ*B, IL-6, and MT2A in the epidermis of diabetic foot skin tissues. (a) Immunohistochemical analysis of NF-*κ*B, IL-6, and MT2A in the diabetic foot skin tissues. The yellowish-brown color denotes the positive areas. Scale bar: 100 *μ*m. The quantitative analyses of NF-*κ*B (b), IL-6 (c), and MT2A (d) expression were performed through measuring the mean density. ^∗^*P* < 0.05, ^∗∗^*P* < 0.01.

The results of immunohistochemical staining clearly showed that NF-*κ*B and IL-6 were expressed in the skin tissues of both AW and CW groups. NF-*κ*B was mainly expressed in some squamous epithelial cells and peripheral inflammatory cells, and its expression was significantly increased in the CW group (*t* = −2.221, *P* = 0.044). IL-6 was principally expressed in the tissues surrounding the inflammatory cells in the dermal layer, while its expression in the CW group was increased (*t* = −2.263, *P* = 0.036). In addition, MT2A was obviously expressed in the cytoplasm of epithelial squamous cells within the AW group, but its expression was remarkably decreased in the CW group (*t* = 4.979, *P* < 0.001) (Figure 3).

### 3.5. Western Blot

**Figure 4:**
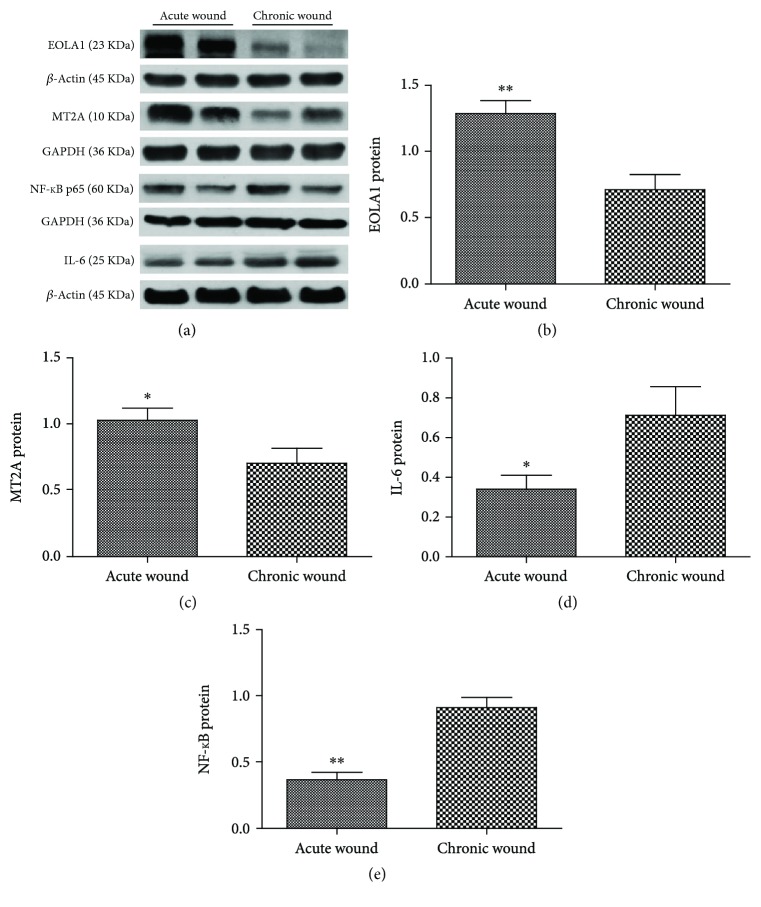
Expression of EOLA1, NF-*κ*B, IL-6, and MT2A in the diabetic wound tissues by western blot (a) and ratios of EOLA1/*β*-actin (b), NF-*κ*B P65/GAPDH (c), IL-6/GAPDH (d), and MT2A/*β*-actin (e) in the wounds from the two groups. ^∗^*P* < 0.05, ^∗∗^*P* < 0.01.

The results of protein electrophoresis revealed that compared with the AW group, the expression of EOLA1 (*t* = 3.817, *P* = 0.001) and MT2A (*t* = 2.307, *P* = 0.033) in the skin tissues was obviously decreased in the CW group, while that of NF-*κ*B (*t* = −5.584, *P* < 0.001) and IL-6 (*t* = −2.298, *P* = 0.034) was remarkably increased (Figure 4).

## 4. Discussion

Diabetic foot disease is one of the most intractable complications of diabetes mellitus and has become an important cause of nontraumatic amputation. The probability of diabetic patients suffering from diabetic foot ulcers (DFUs) during their lifetime can reach 25%, and the amputation rate for DFU patients in China is up to 21.5%. DFUs not only extend the average length of hospital stay, resulting in a huge economic burden, but also increase the risk of amputation, which seriously affects the quality of life and life expectancy of diabetic patients. The reasons for the susceptibility of DM patients to chronic ulcer are not fully understood. In addition to the influence of abnormal metabolic indicators like high blood glucose, it is worthy of paying close attention to inflammatory cell infiltration in the chronic wounds of DFU regardless of the presence or absence of bacterial infection. Moreover, there is high expression of proinflammatory factors such as IL-1*β*, IL-6, IL-8, and TNF-*α*, featured by the reduced phagocytosis of macrophages, decreased apoptosis of inflammatory cells, and persistent inflammatory response [16].

During the observation on the differential differentiation gene map of human endothelial cells activated by LPS stimulation, we cloned a novel gene with upregulated expression, which was named EOLA1, and its carboxyl terminal contained a homologous functional domain similar to that of activation signal co-integron 1 (ASCH 1) and played a role in assisting the transcriptional regulation [17]. Furthermore, we previously observed that the rejection of the transplanted liver was enhanced after inhibiting the expression of EOLA1 in liver transplantation model mice [18], and EOLA1 expression was detected in the heart, skeletal muscles, kidneys, liver, and placenta. In this study, we proved that EOLA1 also existed in human skin and was located in cells. Meanwhile, we found that when the baseline level of systemic inflammation (HsCRP, PCT, IL-6, WBC, and NE%) and the important factors affecting ulcer healing (ABI, hemoglobin, and albumin) were consistent between the two groups, EOLA1 was expressed highly in the skin tissues of the acute wound in DFU but lowly in those of the chronic wound. On the contrary, the expression of some important molecules (e.g., NF-*κ*B and IL-6) in inflammatory activation pathways was higher in the CW group than in the AW group, which is consistent with the previous studies [8, 9]. This suggests that EOLA1 may be involved in the negative regulation of local chronic inflammatory response in DFU.

Our preliminary study showed that the negative regulation of EOLA1 on inflammation was achieved through the interaction with MT. MT is a metal-binding protein with a low molecular weight and expressed in a variety of organs including skin tissues. However, its physiological functions are not fully understood. The mRNA expression of MT was increased after skin irritation and damage [19, 20], indicating that MT can promote the proliferation of epidermal keratinocytes. In the present study, we found that the expression of MT2A was consistent with that of EOLA1; namely, MT2A was highly expressed in the skin tissues of the acute wound in DFU, but its expression was significantly decreased in those of the chronic wound. The results of immunohistochemistry revealed that the expression sites of EOLA1 and MT2A in skin tissues were similar, i.e., the epithelial tissues near the basal cells. In our preliminary study on the function of EOLA1, we observed a significant protein-protein interaction between EOLA1 and MT2A by yeast double hybridization and coimmunoprecipitation, and the results of immunofluorescence detection showed that both EOLA1 and MT2A were colocated in the cytoplasm and nucleus. The above study findings strongly indicate that the interaction between EOLA1 and MT2A may be an important mechanism for inhibiting inflammatory response. As the expression of EOLA1 and MT2A is decreased, DFU presents a persistent inflammatory state and cell proliferation is slowed down, which eventually leads to refractory ulcers.

NF-*κ*B signal activation is mainly mediated by Toll-like receptors (TLRs). The studies have shown that TLRs, dominantly TLR4/9, also participate in the immune inflammatory response of the DFU wound. In the activation of the NF-*κ*B signaling pathway, TLR4 is induced by the cell wall component of pathogenic microorganisms (LPS) and HSPs released in damaged tissue, while TLR9 is mediated by CpG and mitochondrial DNA released in damaged tissue [21, 22]. The activated NF-*κ*B signaling pathway can induce the activation of inflammatory factors such as IL-6 and TNF-*α*[23] and mediate the inflammatory response, cell proliferation, cell differentiation, and other pathophysiological processes [24, 25]. In the present study, the expression of NF-*κ*B, P65, and IL-6 in the skin tissues of DFU was significantly increased in the CW group, suggesting that the inflammatory pathway of the chronic wound is kept activated, and the phenotype of macrophages cannot be transformed from proinflammatory M1 to prohealing M2, causing chronic inflammation and then chronic ulcer. However, how to close the NF-*κ*B inflammatory pathway has not been completely clarified yet. The studies have revealed that MT2A-knockout mice exhibit the obvious activation of the NF-*κ*B signaling pathway and the enhancement of immunoreactivity, indicating that MT2A can negatively regulate immunoreaction-mediated cellular inflammatory response [26]. Therefore, EOLA1 may exert an inhibitory effect on the NF-*κ*B signaling pathway by interacting with MT2A. Furthermore, we have constructed EOLA1-knockout mice to further explore its specific mechanism.

## Figures and Tables

**Table 1:** Demographics and laboratory results of diabetic foot patient cohorts.

Patients (*n*)	Acute wound	Chronic wound	*χ* ^2^ or *t* value	*P* value
	38	41		
Female/male	8/30	12/29	0.70	0.40^#^
Age (years)	62.61 ± 12.31	65.95 ± 11.25	-1.26	0.21
Wagner 2 (*n*/%)	15/39.47	9/21.95		
Wagner ≥ 3 (*n*/%)	23/60.53	32/78.05	2.86	0.09^#^
Average hospitalization time (days)	24.63 ± 15.81	34.71 ± 24.77	-2.17	0.03
Surgical treatment (*n*)	15/39.47	19/46.34	0.38	0.54^#^
Debridement	2	3		
Polymethylmethacrylate	4	2		
Digital or ray amputation	9	11		
Transtibial amputation	0	3		
Vascular diseases (*n*/%)	26/68.42	33/80.49	1.52	0.22^#^
Neuropathy (*n*/%)	28/73.68	33/80.49	0.52	0.47^#^
Retinopathy (*n*/%)	10/26.32	10/24.39	0.04	0.84^#^
Nephropathy (*n*/%)	17/44.74	9/21.95	4.63	0.03^#^
Positive culture (*n*/%)	30/78.95	31/75.61	0.13	0.72^#^
Gram-negative bacteria (*n*/%)	13/34.21	21/51.22		
Gram-positive bacteria (*n*/%)	17/54.84	10/24.39	3.81	0.15^#^
HbA1c < 8% (*n*/%)	11/28.95	17/41.46		
HbA1c ≥ 8% (*n*/%)	27/71.05	24/58.54	1.35	0.25^#^
HsCRP	9.64 ± 5.37	9.29 ± 5.24	0.25	0.80
PCT	1.14±2.66	0.12±0.16	1.48	0.16
IL-6	20.53 ± 17.47	20.35 ± 14.61	0.04	0.97
White blood cells, WBC (10^9^/L)	8.86 ± 4.81	9.52 ± 5.97	-0.54	0.59
Neutrophil percentage (%)	70.17 ± 15.00	68.70 ± 16.45	0.41	0.68
Albumin (g/L)	32.75 ± 4.49	32.46 ± 5.16	0.19	0.85
Hemoglobin (g/L)	115.42 ± 18.11	109.39 ± 19.91	1.41	0.16
Ankle-brachial index, ABI			2.56	0.28^#^
0.9-0.99 (critical value)	9	6		
1.0-1.29 (normal range)	26	34		
>1.3 (arteriosteogenesis)	3	1		

^#^ *χ* ^2^ test. *t*-test was applied to all other parameters.

## Data Availability

All data created during this research are openly available and provided in full in Results of this article.

## References

[1] American Diabetes Association (2014). Standards of medical care in diabetes—2014. *Diabetes Care*.

[2] Tang Y., Zhang M. J., Hellmann J., Kosuri M., Bhatnagar A., Spite M. (2013). Proresolution therapy for the treatment of delayed healing of diabetic wounds. *Diabetes*.

[3] Wu Q. (2018). Hyperbaric oxygen for treatment of diabetic foot ulcers: love you more than I can say. *Annals of Translational Medicine*.

[4] Fabbro M. D., Bortolin M., Taschieri S., Ceci C., Weinstein R. L. (2016). Antimicrobial properties of platelet-rich preparations. A systematic review of the current pre-clinical evidence. *Platelets*.

[5] Wu Q., Lei X., Chen L. (2018). Autologous platelet-rich gel combined with *in vitro* amplification of bone marrow mesenchymal stem cell transplantation to treat the diabetic foot ulcer: a case report. *Annals of Translational Medicine*.

[6] Wu Q., Chen B., Liang Z. (2016). Mesenchymal stem cells as a prospective therapy for the diabetic foot. *Stem Cells International*.

[7] Liang Z., Yang Z. (2004). Identification and characterization of a novel gene EOLA1 stimulating ECV304 cell proliferation. *Biochemical and Biophysical Research Communications*.

[8] Liu Y., Liu H., Chen W., Yang T., Zhang W. (2014). EOLA1 protects lipopolysaccharide induced IL-6 production and apoptosis by regulation of MT2A in human umbilical vein endothelial cells. *Molecular and Cellular Biochemistry*.

[9] Leng W., Lei X., Meng H., Ouyang X., Liang Z. (2015). EOLA1 inhibits lipopolysaccharide-induced vascular cell adhesion molecule-1 expression by association with MT2A in ECV304 cells. *International Journal of Inflammation*.

[10] Liang Z. W., Yang Z. C., Chen J., Luo X. F., Wang X. M. (2007). The effect of inhibiting EOLA1 expression in ECV304 cells. *Chinese Journal of Medical Genetics*.

[11] Satoh M. (2004). Toxicological significance of metallothionein on environmental harmful factors: verification and suggestions from a metallothionein-I/II null mouse model study. *Nippon Eiseigaku Zasshi (Japanese Journal of Hygiene)*.

[12] Coyle P., Philcox J. C., Carey L. C., Rofe A. M. (2002). Metallothionein: the multipurpose protein. *Cellular and Molecular Life Sciences*.

[13] Ma C., Li L. F., Chen X. (2011). Expression of metallothionein-I and II in skin ageing and its association with skin proliferation. *British Journal of Dermatology*.

[14] Holt R. I. G., Cockram C. S., Flyvbjerg A. (2010). *Lack of Metallothionein-I and -II Exacerbates the Immunosuppressive Effect of Ultraviolet B Radiation and Cis-Urocanic Acid in Mice. Textbook of Diabetes*.

[15] Arya A. K., Tripathi R., Kumar S., Tripathi K. (2014). Recent advances on the association of apoptosis in chronic non healing diabetic wound. *World Journal of Diabetes*.

[16] Wicks K., Torbica T., Mace K. A. (2014). Myeloid cell dysfunction and the pathogenesis of the diabetic chronic wound. *Seminars in Immunology*.

[17] Liu Y. M., Liu Z. L., Liu Y. L., Xu X. F., Zhang P., Ma B. (2010). Bioinformatics analysis of human novel gene EOLA1. *Journal of Chengdu Medical College*.

[18] Ran C. F., Dou K., Liang Z., Liu Y., Li K. (2008). Changes in the expression of endothelial-overexpressed lipopolysaccharide-associated factor 1 in grafts during acute rejection following liver transplantation in rats. *The Journal of International Medical Research*.

[19] Jasani B., Anstey A., Marks R., Long C. C., Pearse A. D. (1993). Wild type p53 and metallothionein are expressed simultaneously in UV irradiated skin: a possible link to photocarcinogenesis. *The Journal of Investigative Dermatology*.

[20] Anstey A., Marks R., Long C. (1996). In vivo photoinduction of metallothionein in human skin by ultraviolet irradiation. *The Journal of Pathology*.

[21] Singh K., Agrawal N. K., Gupta S. K., Sinha P., Singh K. (2016). Increased expression of TLR9 associated with pro-inflammatory S100A8 and IL-8 in diabetic wounds could lead to unresolved inflammation in type 2 diabetes mellitus (T2DM) cases with impaired wound healing. *Journal of Diabetes and its Complications*.

[22] Dasu M. R., Martin S. J. (2014). Toll-like receptor expression and signaling in human diabetic wounds. *World Journal of Diabetes*.

[23] Li C., Yang Z., Zhai C. (2010). Maslinic acid potentiates the anti-tumor activity of tumor necrosis factor alpha by inhibiting NF-kappaB signaling pathway. *Molecular Cancer*.

[24] Sánchez Vallecillo M. F., Minguito de la Escalera M. M., Aguirre M. V. (2015). A liquid crystal of ascorbyl palmitate, used as vaccine platform, provides sustained release of antigen and has intrinsic pro-inflammatory and adjuvant activities which are dependent on MyD88 adaptor protein. *Journal of Controlled Release*.

[25] Gehrke N., Garcia-Bardon D., Mann A. (2015). Acute organ failure following the loss of anti-apoptotic cellular FLICE-inhibitory protein involves activation of innate immune receptors. *Cell Death and Differentiation*.

[26] Mita M., Satoh M., Shimada A. (2008). Metallothionein is a crucial protective factor against helicobacter pylori-induced gastric erosive lesions in a mouse model. *American Journal of Physiology-Gastrointestinal and Liver Physiology*.

